# Genome-Wide Analysis of the Glucose-6-Phosphate Dehydrogenase Family in Soybean and Functional Identification of *GmG6PDH2* Involvement in Salt Stress

**DOI:** 10.3389/fpls.2020.00214

**Published:** 2020-02-26

**Authors:** Ying Zhao, Yifan Cui, Shiyu Huang, Jingyao Yu, Xinyu Wang, Dawei Xin, Xin Li, Yonghui Liu, Yuxin Dai, Zhaoming Qi, Qingshan Chen

**Affiliations:** ^1^College of Agriculture, Northeast Agricultural University, Harbin, China; ^2^Key Lab of Maize Genetics and Breeding, Heilongjiang Academy of Agricultural Sciences, Harbin, China

**Keywords:** glucose-6-phosphate dehydrogenase, expression, enzyme activity, salt stress, transgenic plants, soybean (*Glycine max* L.)

## Abstract

Glucose-6-phosphate dehydrogenase (G6PDH) is known as a critical enzyme responsible for nicotinamide adenine dinucleotide phosphate (NADPH) generation in the pentose phosphate pathway (PPP), and has an essential function in modulating redox homeostasis and stress responsiveness. In the present work, we characterized the nine members of the G6PDH gene family in soybean. Phylogenic analysis and transit peptide prediction showed that these soybean G6PDHs are divided into plastidic (P) and cytosolic (Cy) isoforms. The subcellular locations of five GmG6PDHs were further verified by confocal microscopy in *Arabidopsis* mesophyll protoplasts. The respective *GmG6PDH* genes had distinct expression patterns in various soybean tissues and at different times during seed development. Among them, the *Cy*-*G6PDHs* were strongly expressed in roots, developing seeds and nodules, while the transcripts of *P-G6PDHs* were mainly detected in green tissues. In addition, the activities and transcripts of *GmG6PDHs* were dramatically stimulated by different stress treatments, including salt, osmotic and alkali. Notably, the expression levels of a cytosolic isoform (GmG6PDH2) were extraordinarily high under salt stress and correlated well with the G6PDH enzyme activities, possibly implying a crucial factor for soybean responses to salinity. Enzymatic assay of recombinant *GmG6PDH2* proteins expressed in *Escherichia coli* showed that the enzyme encoded by *GmG6PDH2* had functional NADP^+^-dependent G6PDH activity. Further analysis indicated overexpression of *GmG6PDH2* gene could significantly enhance the resistance of transgenic soybean to salt stress by coordinating with the redox states of ascorbic acid and glutathione pool to suppress reactive oxygen species generation. Together, these results indicate that *GmG6PDH2* might be the major isoform for NADPH production in PPP, which is involved in the modulation of cellular AsA-GSH cycle to prevent the oxidative damage induced by high salinity.

## Introduction

The pentose phosphate pathway (PPP) is a pivotal carbohydrate metabolic pathway that acts as a key role in plant development and stress responses (Krüger et al., 2011; Caretto et al., 2015). The PPP is one of the major sources of nicotinamide adenine dinucleotide phosphate (NADPH), which is the principal reducing molecule used in many metabolic pathways, such as nitrogen assimilation and amino acids synthesis (Devi et al., 2007; Sharkey and Weise, 2016). The two dehydrogenases in the PPP pathway, 6-phosphogluconate dehydrogenase (6PGDH, EC 1.1.1.44) and glucose-6- phosphate dehydrogenase (G6PDH, EC 1.1.1.49), utilize NADP^+^ as a cofactor to generate NADPH during the conversion of glucose-6-phosphate (G6P) to pentoses (Wood, 1986). The step catalyzed by G6PDH enzyme is known as the vital reaction in the PPP due to its strict control of the NADPH/NADP^+^ redox balance.

Genes encoding G6PDH have been cloned and characterized from some plants including *Oryza sativa* (Zhang et al., 2013), *Populus suaveolens* (Lin et al., 2005), *Arabidopsis thaliana* (Wakao and Benning, 2005), *Solanum tuberosum* (Wendt et al., 2000; Hauschild and von Schaewen, 2003), *Hordeum vulgare* (Esposito et al., 2001; Cardi et al., 2015), and *Triticum aestivum* (Nemoto and Sasakuma, 2000), and their involvement in plant development has been reported. Cytosolic (Cy) and plastidic (P) isoforms have been certified for plant G6PDHs based on their subcellular localization (Cardi et al., 2013; Castiglia et al., 2015). In addition, the P-G6PDHs are divided into two types, P1-G6PDH and P2-G6PDH, which can be distinguished by diverse gene expression profiles as well as specific biochemical characteristics, indicating the different functions of each isoform in plant metabolism (Cardi et al., 2016). In *Arabidopsis*, there are six G6PDHs targeted to different subcellular compartments: two cytosolic NADP^+^-dependent isoforms encoded by *AtG6PDH5* and *AtG6PDH6* genes respectively, and four plastidic NADP^+^-dependent isoforms encoded by *AtG6PDH1*, *AtG6PDH2*, *AtG6PDH3*, and *AtG6PDH4* genes respectively (Wakao and Benning, 2005). Previous studies have shown that knocking down the cytosolic G6PDH in *Arabidopsis* may inhibit the seed oil accumulation, suggesting that Cy-G6PDH is crucial for regulating the oil biosynthesis during seed development (Wakao et al., 2008). Furthermore, the plastidic isoforms are proved to be essential in providing reducing power (NADPH) for enzymes involved in ammonium assimilation and nitrate reduction (Esposito et al., 2004; Esposito, 2016).

In addition to their pivotal role in developmental processes, the key functions of plant G6PDHs in responses to different types of environmental stresses have been widely proven, including salinity (Wang et al., 2008), cold (Lin et al., 2013), drought (Landi et al., 2016), and heat (Gong et al., 2012). It has been demonstrated that the cytosolic G6PDH is the major contributor to the total cellular G6PDH activity in plants (Castiglia et al., 2015), which seems to be a significant factor for the outcome of abiotic stress responses (Esposito et al., 2001; Honjoh et al., 2007). As a main example, overexpression of a kinetically engineered G6PDH in cytosol enhanced both biotic (defense reactions) and abiotic (drought) stress tolerance of transgenic tobacco through inhibiting NADPH oxidases induced reactive oxygen species (ROS) by improving NADPH provision during early oxidative bursts (Scharte et al., 2009). Besides, overexpression of a cytosolic *PsG6PDH* gene from *Populus suaveolens* confers an increased cold tolerance in transgenic tobacco by elevating the activity of antioxidative enzymes, such as peroxidase and superoxide dismutase, and decreasing the level of membrane lipid peroxidation (Lin et al., 2005, 2013). Also, the enhanced cytosolic G6PDH activities would contribute to the improvement of drought tolerance in soybean roots, with the involvement of ABA-dependent signaling pathway (Liu et al., 2013; Wang et al., 2016).

The characteristics of *G6PDHs* with respect to salt resistance have been validated by several researchers (Liu et al., 2007; Wang et al., 2008; Sang et al., 2018). It has been shown that the oxidative burst is counteracted, more or less, by the activities and expression of G6PDH isoforms upon salt stress (Valderrama et al., 2006; Liu et al., 2007). The Cy-G6PDH isoforms in *Arabidopsis* are essential in the provision of NADPH to maintain the cellular redox homeostasis via the phosphorylation of Thr467 by glycogen synthase kinase 3 under high salinity condition (Dal Santo et al., 2012); and this process is identified as associated with a sugar-signaling molecule (Lejay et al., 2008). Northern hybridization revealed a noticeable increase of *TaG6PDH* transcripts within 12 h of exposure of wheat seedlings to salinity treatment (150 mM NaCl), and the high transcript level was maintained for several hours (Nemoto and Sasakuma, 2000, 2002). In addition, the G6PDH enzyme in rice is the key in sustaining ROS homeostasis by regulating the coordination state of G6PDH activity and NAPDH oxidase under salt stress, however, the molecular metabolism have not been investigated (Zhang et al., 2013).

Although the biological functions of G6PDH in stress responses have been described in several model plants, few information are known about soybean. Here, we characterized nine members of the *G6PDH* gene family in soybean. The cellular locations of *GmG6PDHs* were initially predicted by transit peptide analysis and subsequently verified by transient expression of GFP-tagged GmG6PDH fusion proteins in *Arabidopsis* protoplasts. We also determined the transcriptional profiles of *GmG6PDHs* in distinct organs and under various abiotic stress using qRT-PCR and high-throughput sequencing data analyses. Most notably, one cytosolic isoform (*GmG6PDH2*) had apparent transcriptional response to salt stress and did well correlate with the activity of G6PDH enzyme, possibly implying a prominent role for this isoform in response to salinity. The prokaryotic expression of *GmG6PDH2* in *Escherichia coli* demonstrated that this gene encoded an active G6PDH enzyme. In addition, overexpression of *GmG6PDH2* in soybean hair roots increased the salt tolerance in transgenic soybean seedlings, with higher AsA/DHA (ascorbic acid/dehydroascorbate), GSH/GSSG (reduced/oxidized glutathione) ratios, lower levels of ROS and lipid peroxidation. These findings indicate that the soybean *G6PDHs* participate in plant growth and stress responses, of which the cytosolic *GmG6PDH2* is the main isoform in regulating the cellular redox pool and defending against oxidative stress.

## Materials and Methods

### Identification of *G6PDH* Gene Family in Soybean

To obtain all *G6PDHs* from the soybean genome, a systematic BLASTP search was carried out against the soybean genetics and genomics database (SoyBase^1^) using the published *A. thaliana* G6PDHs as queries. The protein sequences of putative soybean G6PDH family members with an E-value of <10^–10^ and a sequence identity threshold > 90% were downloaded. The candidate genes were further verified by SMART^2^ and Pfam^3^ databases to confirm the presence of a C-terminal NADP-dependent G6PD domain (PF02781) and an N-terminal NADP^+^-binding domain (PF00479). Information about the genetic characteristics of *GmG6PDHs*, including coding sequence lengths, chromosome locations and protein lengths were collected from the SoyBase. The isoelectric point and molecular mass were determined on ExPASy server^4^. Transit peptides and subcellular localization were predicted using TargetP 2.0^5^ and CELLO 2.5^6^ (Yu et al., 2004).

### Evolutionary, Gene Structure, and Synteny Analyses of GmG6PDHs

The full-length proteins of G6PDHs from *Glycine max* (GmG6PDHs), *Zea mays* (ZmG6PDHs), *Oryza sativa* (OsG6PDHs), *Phaseolus vulgaris* (PvG6PDHs), *Medicago truncatula* (MtG6PDHs), *Sorghum bicolor* (SbG6PDHs), *Brachypodium distachyon* (BdG6PDHs), and *A. thaliana* (AtG6PDHs) were used for building a phylogenetic tree using MEGA 5.0 software based on neighbor-joining method with the default parameter values (Tamura et al., 2011). The gene structures of *G6PDHs* were affirmed using the GSDS database^7^ by aligning the coding regions with their corresponding genomic regions. The genomic sequences and coding sequences of *G6PDH* genes in *G. max* and *A. thaliana* were obtained from the soybean genetics and genomics database and NCBI database. The syntenic blocks among *G. max*, *Z. mays*, *A. thaliana*, *O. sativa*, *P. vulgaris*, *M. truncatula*, *B. distachyon*, and *S. bicolor G6PDHs* were identified from the plant genome duplication database (PGDD^8^) (Lee et al., 2012). The gene ID and other information of the *G6PDHs* used in this study were available in Supplementary Table S1.

### Promoter Analysis of *GmG6PDHs*

To investigate the critical *cis*-acting elements in the promoter of *GmG6PDH* genes, 2.0 kb upstream of the position of the ATG codon in these genes were obtained from the soybean genetics and genomics database^9^. The *cis*-acting regulatory DNA elements were predicted from the PlantCARE database^10^ and presented by the IBS 2.0 (Liu et al., 2015).

### Subcellular Localization

The entire coding region of five *GmG6PDH* genes were amplified from the seeds of soybean cultivar “SN14” (provided by the Soybean Breeding Research Center of Northeast Agricultural University, Haerbin, China) by reverse transcription- polymerase chain reaction (RT-PCR) with the high-fidelity KOD*-*Plus-DNA polymerase (TOYOBO, Osaka, Japan). These genes were further constructed into pBI121 vector, which both contain a CaMV35S promoter and green fluorescent protein (GFP) tag. The gene-specific primers used for cloning the putative *G6PDH* genes were shown in Supplementary Table S2. The fusion proteins *pBI121- GmG6PDHs:GFP* or positive control (empty vector) were temporarily expressed in *Arabidopsis* mesophyll protoplasts, which were isolated from the leaves of 14 days-old seedlings grown under a weak light condition to minimize the chloroplast autofluorescence. The subcellular location of GmG6PDH-GFP proteins was monitored 14 h after polyethylene glycol (PEG)-mediated protoplast transfection protocol (Yoo et al., 2007). Confocal laser-scanning microscopy (LSM 710, Carl Zeiss, Jena, Germany) with a 488-nm argon ion laser (for GFP excitation) was used to visualize and localize GFP-tagged proteins. The excitation/emission wavelength were as follows for GFP (488 nm/507 to 535 nm) and chlorophyll autofluorescence (610 nm/650 to 750 nm).

### Expression Analysis of *GmG6PDHs*

The transcriptional patterns of *GmG6PDHs* in multiple tissues via high-throughput sequencing data from Phytozome database^11^, including leaves, root, root hairs, shoot apical meristem, nodules, stem, seed, pod, and flower tissues. The results are shown as heat maps with hierarchical clustering using the software TBtools 0.665 (Chen et al., 2018) and the values were log2-transformed with normalization. To analyze the transcriptional profiles of the *GmG6PDHs* in different stages of seed development, total RNA was extracted from soybean seeds at 4, 7, 14, 30, 50, 80, 110, and 120 days after flowering (DAF). The expression level of *GmG6PDHs* in developing seeds at 4DAF was used as a calibrator. To examine the transcriptional profiling of *GmG6PDHs* under various abiotic stresses, soybean seedlings at the second trifoliolate stage were subjected to salt stress induced by 150 mM NaCl, alkali stress induced by 100 mM NaHCO_3_, and osmotic stress induced by 20% (w/v) PEG (with a molecular weight of 6000 g/M) or 200 mM mannitol solutions. The osmotic potential of 20% PEG6000 and 200 mM mannitol was −0.53 and −0.50 MPa, respectively. Total RNA was extracted from leaf samples at 0, 6, and 12 h after the above treatments. The transcripts of *GmG6PDHs* in soybean leaf under normal environment condition were used as a calibrator. *GmGAPDH* and *GmACTIN* were used as internal reference. Each quantitative real time-polymerase chain reaction (qRT-PCR) reaction was performed in triplicate (technical replicates) on three biological replicates and the transcriptional level of *GmG6PDHs* was calculated based on the 2^–ΔΔ*ct*^ method. All the primers used for qRT-PCR were available in Supplementary Table S2.

### Recombinant Protein Expression and Enzyme Kinetic Property Assay

The CDS of *GmG6PDH2* with the *Xho*I and *Nco*I sites was inserted into the prokaryotic expression vector pET32a (+). The recombinant plasmid *pET32a- GmG6PDH2* was transformed into *E. coli* Rosetta strain to produce the putative recombinants. The positive clone was sequenced and cultivated in liquid LB medium supplemented with 1 mM IPTG at 37°C for 4 h to induce the expression of *GmG6PDH2*. The recombinant proteins were wall-broken by ultrasonic wave with a power output of 250 W for 10 min and then harvested by centrifuging at 10,000 *g* for 15 min. The supernatant was detected by 12% SDS-PAGE and collected for enzymatic activities assay. The total protein concentration was monitored by Bradford Protein Assay Kit purchased from Solarbio Science and Technology (Beijing, China). The kinetic parameters of GmG6PDH2 recombinant proteins with regard to the glucose-6-phosphate (G6P) and NAPD^+^ was determined using Eadie–Hofstee plot (Wakao and Benning, 2005).

### G6PDH Activity Assays

G6PDH activity assays were performed as described by Wakao and Benning (2005), with slight modifications. The soybean roots (0.2 g) were extracted in 5 mL extraction buffer containing 50 mM 2-[4-(2-Hydroxyethyl)-1-piperazinyl]ethanesulfonic acid -Tris(hydroxymethyl)aminomethane (Hepes-Tris) buffer (pH 7.8), 1 mM EDTA, 3 mM MgCl_2_ and 1 mM phenylmethylsulfonyl fluoride. The G6PDH activities were measured with regard to the oxidation of G6P by NADP^+^. The total reaction mixture was reduced to 1 mL with 0.5 mM NADPNa_2_, 0.5 mM D-glucose-6-phosphate disodium salt, 3.3 mM MgCl_2_, 50 mM Hepes-Tris (pH 7.8) and an appropriate amount of enzyme extracts (Wakao and Benning, 2005). Hepes-Tris buffer was made as follows: 0.5M Hepes was titrated to pH 7.8 with about 1M Tris, and then diluted 10-fold to give 50 mM Hepes.

### *Agrobacterium*-Mediated Over-Expression of *GmG6PDH2* in Soybean Hairy Roots

The plasmid of *pBI121-GmG6PDH2:GFP* was transformed by electroporation into *Agrobacterium rhizogenes* strain K599, which was used to transform soybean hypocotyls. Soybean transformation in “SN14” hypocotyls and hairy root induction were performed as reported previously (Tóth et al., 2016). Soybean plants infected with the *A. rhizogenes* strain K599 were considered as control hairy roots. The transgenic lines were screened by PCR amplification and enzyme activity assay as described (Pan et al., 2016), and the non-transgenic hair roots were removed from the seedlings. Transgenic lines with similar-length hairy roots were selected and treated with 1/2 Hoagland solution containing 0 or 100 mM NaCl for 5 days, respectively. The root fresh weight and maximum root length of transgenic soybean plants were researched after 5 day of salt treatment. More than 10 independent hairy root lines were analyzed in this work to check the effects of *GmG6PDH2* over expression on salinity stress responses.

### Analysis of Cellular ROS Levels and Antioxidant Contents

The metabolites contents of AsA-GSH cycle, including AsA and GSH, and their oxidized forms, DHA and GSSG were determined using Ascorbic Acid or Glutathione Colorimetric Assay Kit purchased from Solarbio Science and Technology (Beijing, China), as per the manufacturer’s protocol. The assays of hydrogen peroxide (H_2_O_2_) content were conducted according to the method published previously (Velikova et al., 2000). The membrane damage was determined with regard to thiobarbituric acid- reactive substances (TBARS) content, a product of lipid peroxidation (Hodges et al., 1999). Briefly, the soybean root samples (0.5 g) were extracted with 10 mL 0.1% (w/v) trichloroacetic acid (TCA), and then the homogenate was centrifuged at 10,000 *g* for 10 min at 4°C. The supernatant was used for the determination of H_2_O_2_ and TBARS contents. The total reaction volume of H_2_O_2_ assay was 2 mL containing 0.5 mL of the supernatant, 1 mL 1M potassium iodide and 0.5 mL 10 mM potassium phosphate buffer (pH 7.0). The intensity was measured at 390 nm. The total reaction volume of TBARS assay was 2 mL containing 0.5 mL of the supernatant and 1.5 mL 0.5% (w/v) thiobarbital acid in 15% TCA. The absorbancy of supernatant was read at both 532 and 600 nm.

### Statistical Analysis

All experiments were performed with at least three biological replicates. Values are presented as mean ± SD. The significance of the data was evaluated using Student’s *t*-test with SPSS statistics 22.0 software. The significance level was set at *P* < 0.05.

## Results

### Identification and Classification of *G6PDH* Gene Family in Soybean

In this study, full-length proteins and conserved domains of six glucose-6-phosphate dehydrogenases (G6PDHs) in *A. thaliana* were used as BLAST queries against the soybean genetics and genomics database^12^. A total of nine *G6PDH* genes were originally obtained in the soybean genome, which were designated as *GmG6PDH1-9* (Table 1). Full-length coding sequences of *GmG6PDH 1-9* ranged from 1560 to 1839 bp, and encoded nine putative proteins with 518 to 612 amino acid residues. The protein isoelectric points and molecular mass of the nine GmG6PDHs varied from 5.80/59.3 to 8.76/68.8 kDa, respectively (Table 1). Online software, CELLO 2.5 and TargetP 1.1, were used to assess the existence of a predicted N-terminal transit peptide (TP), which indicated the localization of GmG6PDH2, 4 and 6 in the cytosol, and others in the plastid (Table 1).

**TABLE 1:** Basic information of the nine soybean *G6PDH* genes (*GmG6PDHs*).

			**ORF length**	**Protein**	**Isoelectric**	**Molecular**	**Subcellular**
**Gene name**	**Gene ID^1^**	**Gene location**	**(bp)**	**length**	**point**	**mass (KDa)**	**localization**
*GmG6PDH1*	Glyma.03G229400.1	Gm3 43144328-43150674	1839	612	6.58	68.8	Plastidic
*GmG6PDH2*	Glyma.19G082300.1	Gm19 29813147-29821693	1557	518	6.32	59.3	Cytoplasmic
*GmG6PDH3*	Glyma.08G199000.1	Gm8 16078525-16083556	1767	588	8.76	66.3	Plastidic
*GmG6PDH4*	Glyma.16G063200.1	Gm16 6210393-6217815	1557	518	5.80	59.3	Cytoplasmic
*GmG6PDH5*	Glyma.02G096800.1	Gm2 8700742-8705334	1809	602	8.28	68.2	Plastidic
*GmG6PDH6*	Glyma.19G077300.1	Gm19 27787739-27797138	1560	519	6.32	59.7	Cytoplasmic
*GmG6PDH7*	Glyma.18G284600.1	Gm18 56525770-56534088	1806	601	7.63	67.9	Plastidic
*GmG6PDH8*	Glyma.07G013800.1	Gm7 1073133-1082652	1767	588	8.03	66.5	Plastidic
*GmG6PDH9*	Glyma.19G226700.1	Gm19 47838450-47844119	1815	604	6.37	68.1	Plastidic

**FIGURE 1:**
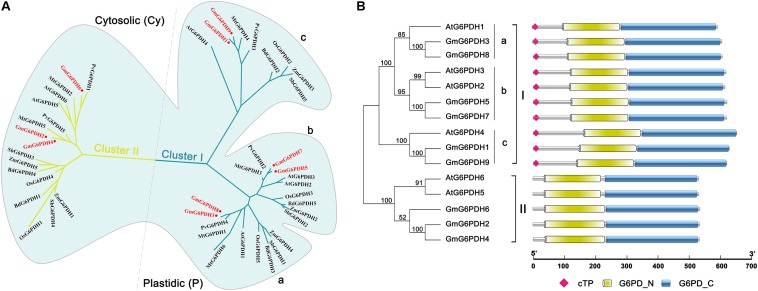
Phylogenic and protein domain analysis of GmG6PDHs. **(A)** Phylogenetic tree of G6PDH proteins from *Glycine max* (red circles), *Arabidopsis thaliana*, *Zea mays*, *Oryza sativa*, *Phaseolus vulgaris*, *Medicago truncatula*, *Brachypodium Distachyon*, and *Sorghum bicolor*. **(B)** The bi-domain structure of the soybean and *Arabidopsis* G6PDH proteins. Predicted chloroplast transit peptide (cTP) is depicted as colored diamonds.

To examine the classification and evolutionary history of soybean G6PDHs, the full-length protein sequences of GmG6PDHs were aligned with homologous G6PDHs from *S. bicolor* (StG6PDH1-4), *A. thaliana* (AtG6PDH1-6), *O. sativa* (OsG6PDH1-5), *Z. mays* (ZmG6PDH1-6), *B. distachyon* (BdG6PDH1-5), *M. truncatula* (MtG6PDH1-7), and *P. vulgaris* (PvG6PDH1-5), and a phylogenetic tree was constructed. As shown in Figure 1A, the phylogenic analysis suggested that the different plant G6PDHs could be clearly classified into two major clades (I and II). Clade I corresponded to plastidic (P) isoforms containing four *Arabidopsis* P-G6PDHs (AtG6PDH1-4) (Wakao and Benning, 2005; Née et al., 2009). Clade I was further segmented into three classes (a, b, and c), in which GmG6PDH3 and GmG6PDH8 were subdivided into class a, along with an *Arabidopsis* P1 isoform (AtG6PDH1); GmG6PDH5, 7 and two *Arabidopsis* P2 isoforms (AtG6PDH2, 3) fall into class b; GmG6PDH1, 9 and an inactive-G6PDH isoform (AtG6PDH4) were clustered within class c. Moreover, clade II corresponded to the cytosolic (Cy) isoforms, including GmG6PDH2, 4 and 6, together with two *Arabidopsis* Cy-G6PDHs (AtG6PDH5, 6) (Wakao et al., 2008). The phylogenetic clades were in accordance with the *in silico* prediction of GmG6PDHs. As expected, the GmG6PDH isoforms were more closely related to its homolog from *P. vulgaris* in each cluster, which all belonged to the leguminous family (Figure 1A).

Structural analysis of translated proteins for *GmG6PDH1-9* demonstrated that the GmG6PDHs exhibited a bi-domain protein structure similar to the reported *A. thaliana* G6PDH proteins, consisting of an N-terminal NADP^+^-binding domain (PF00479) and a C-terminal G6PD domain (PF02781) (Figure 1B). Two motifs, NADP^+^-binding motif (NEFVIRLQP) and substrate-binding motif (RIDHYLGKE), were highly conserved in all the GmG6PDH proteins (Supplementary Figure S1). These protein sequences also contained a conserved Rossman fold (GXXGDLA) domain (Supplementary Figure S1). Based on the subcellular localization prediction, phylogenetic and protein structural analyses, GmG6PDHs were separated into two types: three cytosolic NADP^+^-G6PDH isoforms (GmG6PDH2, 4 and 6), and six plastidic NADP^+^-G6PDH isoforms (GmG6PDH1, 3, 5, 7, 8, and 9). The specific localization feature as represented by soybean *G6PDH* genes revealed the relatedness of distinct function for G6PDH isoforms in each clade with their evolutionary process.

### Syntenic Relationship and Gene Structure Analysis of *GmG6PDHs*

**FIGURE 2:**
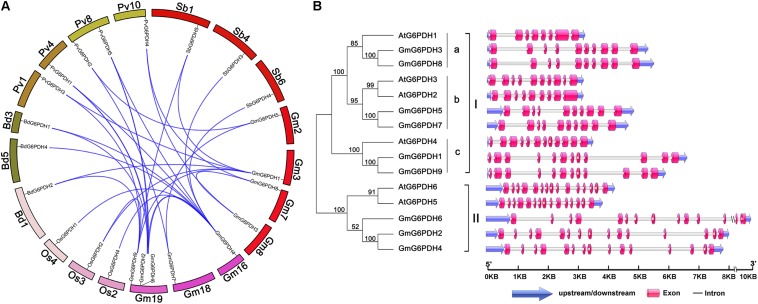
Syntenic and exon–intron structural analysis of *GmG6PDH* family genes. **(A)** Syntenic analysis of soybean *G6PDHs* with the corresponding genes in *B. distachyon*, *P. vulgaris*, *S. bicolor*, and *Oryza sativa*. The chromosome of the above species is shown as a circle. The colored curves indicate the collinear region of the G6PDHs. **(B)** The exon–intron organization of *AtG6PDHs* and *GmG6PDHs*. Untranslated regions (UTRs) are indicated by blue arrow. Exons and introns are visualized as colored boxes and gray lines respectively.

A synteny analysis among *G6PDHs* from *G. max*, *P. vulgaris*, *Z. mays*, *O. sativa*, *A. thaliana*, *M. truncatula*, *B. distachyon*, and *S. bicolor* was performed in the present study to gain some insight into the potential function of *GmG6PDHs*. As shown in Figure 2A, the nine *GmG6PDHs* were scattered along seven out of twenty soybean chromosomes and each of the seven chromosomes comprised one to three *GmG6PDHs*. Moreover, a total of 30 orthologous pairs of *G6PDHs* were found in the above eight species (Supplementary Figure S2 and Supplementary Table S3). The *GmG6PDHs* had syntenic relationship only with *PvG6PDHs*, *OsG6PDHs*, *SbG6PDHs* and *BdG6PDHs*, including four orthologous gene pairs between *G. max* and *O. sativa* or *S. bicolor*, five orthologous gene pairs between *G. max* and *B. distachyon*, and 12 orthologous gene pairs between *G. max* and *P. vulgaris* (Figure 2A). Besides, five paralogous *G6PDH* gene pairs were confirmed in soybean genome, and the paralogous gene pairs were apt to be found among the members in the same subfamily (Figure 2A). To obtain further details about the structural diversity of *GmG6PDH* genes, we subsequently compared the localization and size of exon/intron among *GmG6PDH*s and *AtG6PDHs*. As shown in Figure 2B, *G6PDH* genes belonged to the same cluster shared the similar exon–intron structures, particularly in relation to exon numbers. For instance, *G6PDH* genes in cluster I possess 8–12 exons, and *G6PDH* genes in cluster II exhibited an equal number of exons (15) and nearly identical exons length. These results indicated that the *GmG6PDH* genes were highly conserved in gene sequence and exon–intron organization within each phylogenic group.

### Regulatory Elements in the *GmG6PDH* Promoters

**FIGURE 3:**
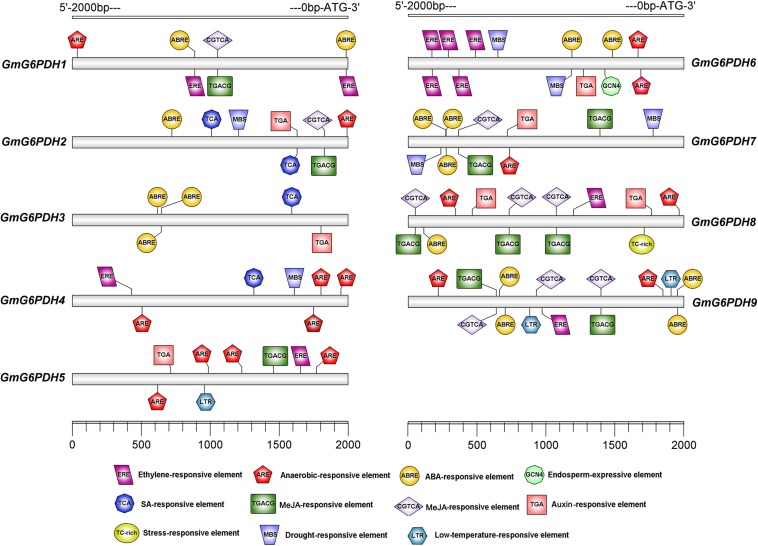
*Cis*-elements prediction in the 2.0 kb promoter region upstream from the start codon of *GmG6PDHs*. The relative positions of *cis*-elements in each *GmG6PDH* gene are marked by different-colored boxes.

To identify putative *cis*-elements involved in *GmG6PDHs* transcriptional regulation, a 2.0 kb promoter region upstream from the ATG translation start codon of each *GmG6PDH* gene was analyzed. As shown in Figure 3, most *GmG6PDH* genes displayed the existence of some stress responsive *cis*-elements, such as ARE, a *cis*-regulatory element involved in anoxic-inducibility, was found in all *GmG6PDH* genes, except for *GmG6PDH3.* Beyond that, MBS, a *cis*-element involved in drought responsiveness, was found in *GmG6PDH2*, *GmG6PDH4*, *GmGPDH6*, and *GmGPDH7* genes; TC-rich repeats, a *cis*-element associated to stress and defense responsiveness, was observed in *GmG6PDH8*; and LTR (cold-responsive element) was seen in *GmG6PDH5* and *GmG6PDH9* genes (Figure 3). Meanwhile, all *GmG6PDH* promoter sequences contained one or more *cis*-elements involved in response to multiple hormones, such as auxin-responsive element (TGA), ABA-responsive element (ABRE), and SA-responsive element (TCA). Remarkably, GCN4, a *cis-*acting element required for endosperm expression was observed in *GmG6PDH6*. Bioinformatics analyses of *cis*-elements suggest *GmG6PDHs* may be pivotal in mediating stress responses as well as plant growth.

### Subcellular Localization of *GmG6PDHs*

**FIGURE 4:**
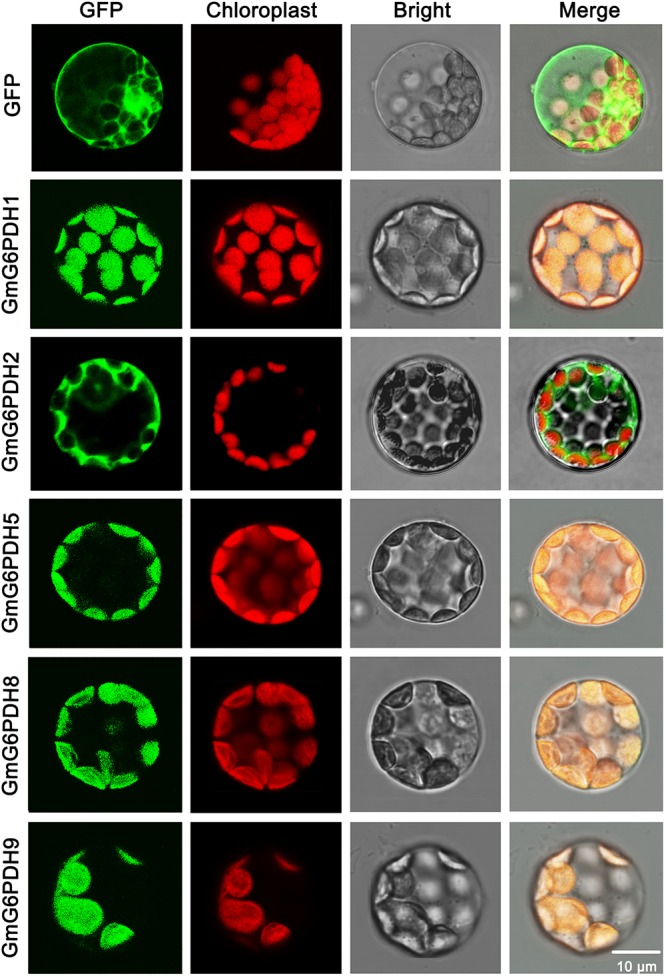
Subcellular localization analysis of GmG6PDHs by transiently expressing five GmG6PDH-GFP fusion proteins in *Arabidopsis* mesophyll protoplasts. Confocal micrographs showing the subcellular localization of GmG6PDH1, GmG6PDH2, GmG6PDH5, GmG6PDH6, and GmG6PDH9 proteins. Merged pictures of the GFP fluorescence (first panels), the chloroplast autofluorescence (second panels) and the corresponding bright field (third panels) are shown in the fourth panels.

The cellular location of G6PDH proteins is closely linked to and indicates their functions. To certify the subcellular localization of GmG6PDHs, the coding sequences of five *GmG6PDHs* (*GmG6PDH1*, *2*, *5*, *8*, and *9*) were successfully cloned, verified and submitted to GenBank with the following accession numbers: *GmG6PDH1* (MN339553), *GmG6PDH2* (MN339554), *GmG6PDH5* (MN339555), *GmG6PDH8* (MN339556), and *GmG6PDH9* (MN339557). We then fused the coding region of five *GmG6PDHs* in-frame with the N-terminus of the GFP coding region. The positive control (35S: GFP) and GFP-tagged GmG6PDH proteins were transiently expressed in *Arabidopsis* mesophyll protoplasts. The free GFP protein was evenly distributed throughout the cell except for chloroplast and vacuole (Figure 4), while the GmG6PDH1, 5, 8 and 9 were specifically targeted to the chloroplast. Moreover, the GmG6PDH2 fusion protein was merely detected in the cytosol, suggesting that it encoded a cytoplasmic protein (Figure 4). These results were in line with the previous online prediction.

### Expression Profiles of *GmG6PDHs* in Different Tissues and Developmental Phases

**FIGURE 5:**
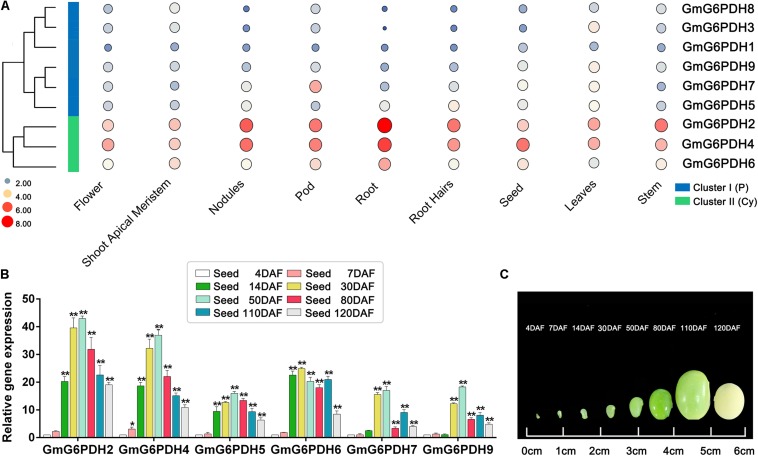
Expression profiles of *GmG6PDHs* in multiple tissues and developmental stages. **(A)** Expression patterns cluster analysis of *GmG6PDHs* involved in tissue development. The transcripts of *GmG6PDH* genes in various tissues are investigated from Phytozome database. The results are shown as heat maps. The color scale represents log2 expression values, with red denoting high-level transcription and blue denoting low-level transcription. The size of the circle also indicates the transcription level, with a larger circle indicating high-abundance transcripts. **(B)** The transcript profiling and **(C)** physiological phenotype of *GmG6PDHs* in developing seeds at 4, 7, 14, 30, 50, 80, 110, and 120 days after flowering (DAF). The transcripts of *GmG6PDHs* in developing seeds at 4 DAF were served as the internal reference. Three biological replicates for each tissue were collected. Asterisks above bars denote a statistically significant difference by Student’s *t*-test (*P < 0.05, **P < 0.01).

In succession, we evaluated the transcriptional patterns of *GmG6PDHs* in multiple tissues *via* high-throughput sequencing data from Phytozome database, including leaves, root, root hairs, shoot apical meristem, nodules, stem, seed, pod, and flower tissues. The transcripts of nine *GmG6PDHs* could be observed in all the tissues tested, but the transcriptional profiles were different between the cytosolic (*GmG6PDH2*, *4*, and *6*) and plastidial (*GmG6PDH1*, *3*, *5*, *7*, *8*, and *9*) isoforms (Figure 5A). The cytosolic *GmG6PDH*s were strongly expressed in roots, pod and nodules. Nonetheless, the transcripts of plastidial *GmGPDH3*, *5*, and *9* genes were primarily seen in leaves, and *GmG6PDH7* gene was mainly expressed in pod. The transcriptional levels of *GmG6PDH1* and *8* genes were comparatively low in all nine tissues compared with other genes. Furthermore, the *GmG6PDHs* were classed into two major groups (I and II) based upon their expression patterns across all nine tissues, which is correlated with the phylogenetic clades for GmG6PDHs (Figure 5A). Within each subgroup, most of these genes exhibited similar expression profiles. Notably, the average relative mRNA levels of cytosolic *GmG6PDHs* in group II were much higher than that of plastidic *GmG6PDHs* in group I. The tissue-specific expression characteristics of *GmG6PDHs* reflected their versatile functions in multiple aspects of soybean growth and development.

In addition, it has been reported that G6PDH enzymes are required for the accumulation of lipid and starch in developing seeds (Wakao et al., 2008). Therefore, to explore the potential function of *GmG6PDHs* during soybean seed development, the transcriptional patterns of *GmG6PDHs* in developing seeds at different stages was further affirmed by qRT-PCR. All the tested genes remained extremely high expression levels during early-middle seed development (14, 30, and 50 DAF) and low expression levels during the late maturation stage of seed development (110 and 120 DAF) (Figure 5B). The maximum level of *GmG6PDH2*, *4*, *5*, *7*, and *9* genes expression occurred in developing seeds at 50 DAF, while the expression level of *GmG6PDH6* was peaked in developing seeds at 30 DAF. Among the tested genes, the transcript abundance of cytosolic *GmG6PDH2* gene was the highest at several stages of seed development (30, 50, 80, 110, and 120 DAF).

### Enzyme Activity and Transcript Level of *GmG6PDHs* Under Abiotic Stress

**FIGURE 6:**
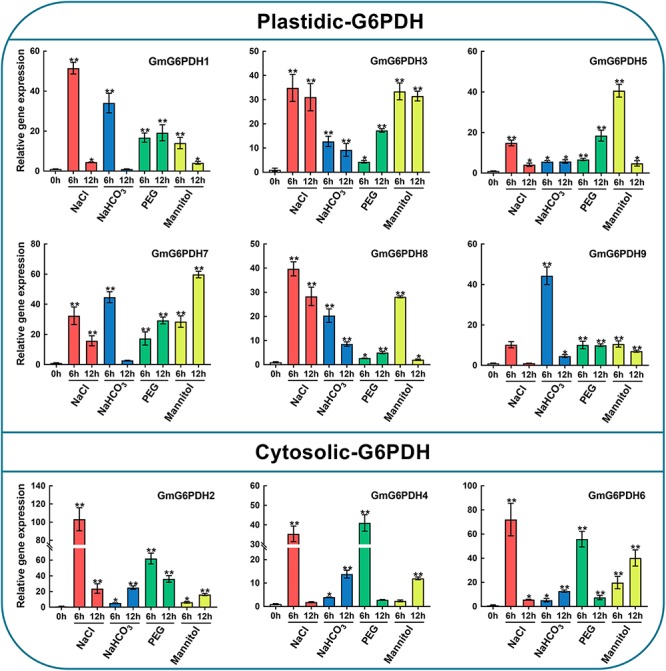
Expression profiles of *GmG6PDHs* in leaves of soybean plants subjected to 150 mM NaCl, 100 mM NaHCO_3_, 20% PEG, 200 mM mannitol or water (control) for 0, 6, and 12 h, respectively. The expression of *GmG6PDHs* in non-stress conditions was used as a calibrator. Three independent biological replicates were carried out and the results of qRT-PCR were analyzed using the 2^–ΔΔct^ method. Asterisks above bars denote a statistically significant difference by Student’s *t*-test (*P < 0.05, **P < 0.01).

It is well-known that G6PDH genes are important for stress adaptation in several model plants (Wakao and Benning, 2005; Long et al., 2016). Also, the promoter analysis of soybean *G6PDHs* revealed a number of potential *cis-*elements and transcription binding motifs (MBS, ABRE, ARE, and TCA-elements) involved in responsiveness to abiotic stresses, such as salt and drought stresses frequently encountered in our area (northeast China). Hence, to further understand how *GmG6PDHs* respond to abiotic stresses, we analyzed the transcriptional profiles of *GmG6PDHs* under alkali (100 mM NaHCO_3_), salt (150 mM NaCl), and osmotic (20% PEG or 200 mM mannitol) stresses. As shown in Figure 6, there were distinct differences among cytosolic and plastidial *GmG6PDH* isoforms based on expression patterns under various stress conditions. The expression levels of cytosolic *GmG6PDHs* (*GmG6PDH2, 4, and 6*) were extraordinarily high upon salt stress, of which the transcript expression of *GmG6PDH2* was highly inducible (reaching upto 100-fold) under the salinity stress treatment for 6 h. The plastidic forms *GmG6PDH1, 3, 7, and 8* were also upregulated under salinity stress of 6 h (about 30–40-fold), while the average mRNA levels of *P-G6PDHs* was much lower than that of *Cy-G6PDHs*. Also, the cytosolic *GmG6PDHs* were dramatically induced at the early stage of PEG treatment, and the transcripts of *GmG6PDH6* at 6 h of treatment were much higher than that of other genes. Furthermore, most of *GmG6PDH* genes were apparently up-regulated under alkaline treatment, particularly the plastidial *GmG6PDHs*, which had obviously high level of transcription at 6 h. In contrast, the cytosolic *GmG6PDHs* showed the maximum transcriptional level at 12 h of the same treatment. Likewise, the plastidial *GmG6PDHs* were originally stimulated after 6 h of mannitol treatment, maintaining comparatively high abundant transcripts throughout the entire treatment period, while the cytosolic *GmG6PDHs* were markedly up-regulated after 12 h (Figure 6).

**FIGURE 7:**
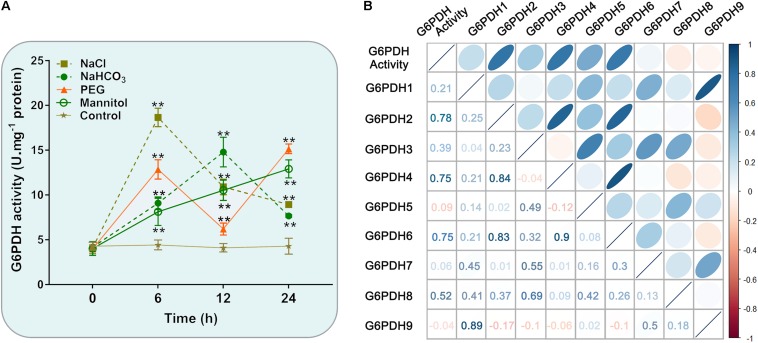
Enzyme activity profiles of G6PDH in soybean under different abiotic stress treatments. **(A)** Analysis of G6PDH activity in leaves of soybean plants subjected to 150 mM NaCl, 100 mM NaHCO_3_, 20% PEG, 200 mM mannitol or water (control) for 0, 6, 12, and 24 h, respectively. Data represent the means ± SD of three biological replicates. Asterisks above bars denote a statistically significant difference from the control group by Student’s *t*-test (*P < 0.05; **P < 0.01). **(B)** Correlation coefficient between G6PDH enzymatic activity and the expression levels of *GmG6PDHs*. Each ellipse chart represents the correlation coefficient between any two traits. The color and slope of the ellipse represent magnitude of correlation. The ellipses of negative correlations are displayed in red and positive correlations in blue.

Similarly, the enzymatic assay revealed that the soybean G6PDH activity was significantly increased under NaCl, NaHCO_3_, PEG and mannitol treatments (Figure 7A). The G6PDH activities under the above treatments were approximately 1.5- to 4.2 -fold higher than that under the normal condition. What’s more, salt treatment exhibited an even higher stimulatory influence on G6PDH activity than other treatments, which causes the rapid increases of the enzyme activity within 6 h after salt stress (Figure 7A). During drought treatment, the G6PDH activity first peaked at 6 h and was raised again during the 12–24 h time period. The level of the G6PDH activity increased and then decreased during alkali treatment, and peaked at 12 h, while the G6PDH activity exhibited a continuous increase during osmotic treatment. Meanwhile, correlation analysis revealed the trend in G6PDH activity under abiotic stresses was well-consistent with gene expression of cytosolic *GmG6PDH2*, which indicated that it to encode the major G6PDH isoform involved in response to abiotic stresses (Figure 7B).

### Overexpression of *GmG6PDH2* Increases Salt Tolerance

**FIGURE 8:**
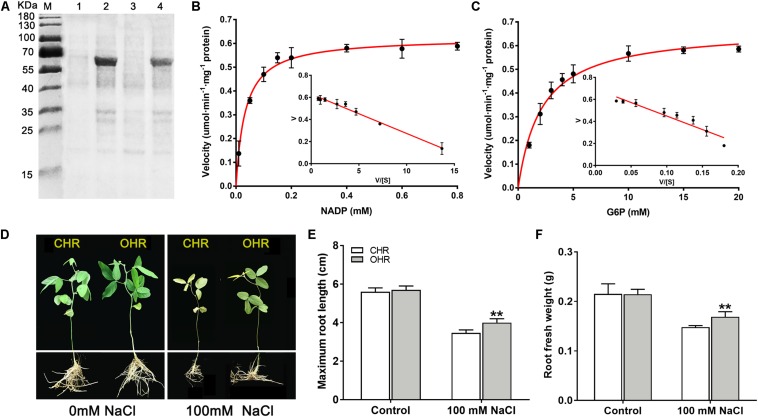
Overexpression of cytosolic NADP^+^-dependent isoform (GmG6PDH2) confers salt tolerance in transgenic soybean. **(A)** Coomassie-stained 12% SDS-PAGE of crude extracts of *Escherichia coli* cells harboring pET32a-GmG6PDH2 without (Lanes 1, 3) and with IPTG induction (Lanes 2, 4). The kinetic properties of *GmG6PDH2* with regard to **(B)** NADP^+^ and **(C)** glucose-6-phosphate by Eadie–Hofstee plot. **(D)** Performance of soybean plants harboring *GmG6PDH2*-overexpressing hairy roots (OHR) and control hairy roots (CHR) treated with 0 or 100 mM NaCl for 5 days. CHR plants are generated after the infection of *Agrobacterium rhizogenes*. Comparisons of **(E)** maximum root length and **(F)** root fresh weight of CHR and OHR plants subjected to 0 or 100 mM NaCl for 5 days. More than 10 hair root lines were investigated and shown as means ± SD. Asterisks above bars denote a statistically significant difference from the CHR plants by Student’s *t*-test (*P < 0.05, **P < 0.01).

To deeply comprehend the resistance functions of *GmG6PDH2* gene, its catalytic characteristics were first determined by extracting the crude proteins from *E. coli* cells expression plasmid of His-tagged *GmG6PDH2*. An expected molecular mass of GmG6PDH2-His fusion protein (consisting of histidine marker and target gene) was identified by SDS/PAGE (Figure 8A). The recombinant GmG6PDH2 protein was analyzed for its enzyme kinetic with regard to both substrates: glucose-6-phosphate (G6P) and NADP^+^. The kinetic parameters were calculated by using Eadie–Hofstee data plots, in which the *K*_m_ of G6P and NADP^+^ was estimated as 2.14 and 0.033 mM respectively, and the *V*_max_ of G6P and NADP^+^ was estimated as 0.67 and 0.62 umol⋅min^–1^⋅mg^–1^ protein respectively (Figures 8B,C). These results demonstrated that the protein encoded by *GmG6PDH2* had the functional NADP^+^-dependent G6PDH enzyme activity.

Next, to better elucidate how *GmG6PDH2* gene contribute to the response to high salinity, *GmG6PDH2*-overexpressing soybean hairy roots (OHRs) were generated by *Agrobacterium rhizogenes*-mediated gene transformation, of which 10 positive transgenic plants were verified by PCR detection (Supplementary Figure S3). The physiological activity test signified that the G6PDH enzyme activity in *GmG6PDH2*-OHR lines were 1.7- to 2.5-fold higher than that in the control hair roots (CHR), which suggested that *GmG6PDH2* gene was expressed successfully and functioned with G6PDH activities in OHR lines (Supplementary Figure S3). To look into the effects of *GmG6PDH2* overexpression on salt tolerance at soybean seedling stage, 4-week-old CHR and OHR plants were transferred to 1/2 Hoagland solution supplemented with 0 or 100 mM NaCl for 5 days. As demonstrated in Figure 8D, the CHR seedlings exhibited a severe growth-inhibitory phenotype, with a partial leaf shriveling phenomenon, whereas *GmG6PDH2*-OHR plants displayed significantly improved resistance to salinity stress, as reflected by a higher root fresh weight and root length (Figures 8E,F).

### *GmG6PDH2* Is Essential for AsA and GSH Biosynthesis Under Salt Stress

**FIGURE 9:**
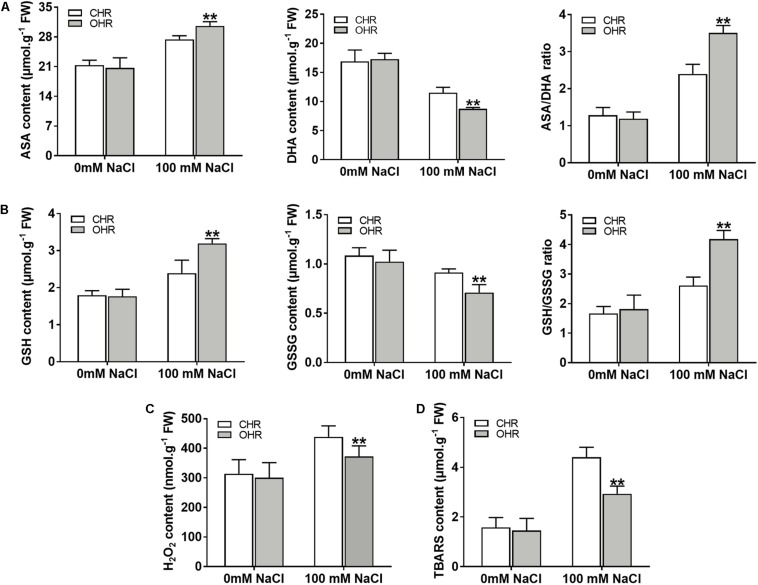
Overexpression of *GmG6PDH2* improves the ROS-scavenging capacity under high salinity condition. **(A)** The contents of AsA and DHA, and the ratio of AsA/DHA; **(B)** The contents of GSH and GSSG, and the ratio of GSH/GSSG; **(C)** H_2_O_2_ and **(D)** TBARS levels in CHR and OHR plants subjected to 0 or 100 mM NaCl for 5 days. More than 10 independent transgenic hairy root lines were analyzed and shown as means ± SD. Asterisks above bars denote a statistically significant difference from the CHR plants by Student’s *t*-test (^∗^*P* < 0.05, ^∗∗^*P* < 0.01).

It has been reported that the plant G6PDHs perform significant functions in maintaining the cellular redox balance (Krüger et al., 2011; Cardi et al., 2016). To validate whether overexpression of cytosolic *GmG6PDH2* gene could affect the redox fluctuation, the redox states of AsA and GSH were monitored in soybean transgenic hair roots. The levels of reduced and oxidized AsA and GSH did not change among the CHR and OHR plants under standard growing conditions (Figures 9A,B). Nevertheless, the *GmG6PDH2*-OHR plants accumulated higher contents of AsA and GSH, and substantially lower GSH and GSSG contents than the CHR plants, thereby resulting in much higher ratios of AsA/DHA and GSH/GSSG. These results illustrated that the function of *GmG6PDH2* gene in salt tolerance could partly ascribe to the improved regeneration of the reduced form of AsA and GSH.

In plant stress reactions, the AsA-GSH cycle has been considered as a powerful ROS scavenging pathway, exclusively eliminating the cellular hydrogen peroxide (H_2_O_2_) through utilizing the AsA and GSH (Fotopoulos et al., 2010; Li et al., 2010). Thus, the remarkable changes in reduced and oxidized forms of ASA and GSH in *GmG6PDH2* transgenic lines reminded us to examine the H_2_O_2_ contents upon salt stress. As expected, the contents of H_2_O_2_ in *GmG6PDH2*-OHR lines were remarkably lower than that in the CHR plants, supporting an important role for *GmG6PDH2* in ROS detoxification under salt stress (Figure 9C). Moreover, the TBARS levels, as an indicator of lipid peroxidation, were evidently lower in OHR plants than that in the CHR plants under salt stress (Figure 9D). In conclusion, these results suggested that overexpression of cytosolic *GmG6PDH2* gene alleviated the stress-induced ROS accumulation through the regulation of the redox status of AsA and GSH, and consequently minimize the cell membrane damages under salt stress.

## Discussion

Soybean as the important economic food crop is one of the major sources of plant protein and oil for mankind, and its growth is largely affected by different abiotic stresses (Ghosh and Islam, 2016). A great number of researches have shown that *G6PDHs* play a critical role in plant growth and abiotic stress responses (Esposito, 2016; Hýsková et al., 2017). Although *G6PDH* genes have been cloned from several model organisms such tobacco (Scharte et al., 2009; Silva et al., 2018), *Arabidopsis* (Wakao and Benning, 2005), barley (Cardi et al., 2013; Caretto et al., 2015), wheat (Nemoto and Sasakuma, 2000) and tomato (Landi et al., 2016), there is scarce information about their biological functions in soybean. In this study, we identified nine *G6PDH* gene family members from the soybean genome, named as *GmG6PDH1-9* (Table 1). Similar to other reported G6PDHs, all the GmG6PDH proteins contained the typical and necessary protein domains (PF00479, PF02781) (Figure 1B). Meanwhile, *GmG6PDH* genes have two highly conserved motifs in their translated protein sequences: NADP^+^-binding motif (NEFVIRLQP) and substrate- binding motif (RIDHYLGKE) (Supplementary Figure S1), illustrating the proteins encoded by *GmG6PDH1-9* have enzyme catalytic activities (Hauschild and von Schaewen, 2003). According to the putative subcellular location, GmG6PDHs were segmented into two types: three cytosolic (Cy) isoforms (GmG6PDH2, 4, and 6), six plastidic (P) isoforms (GmG6PDH1, 3, 5, 7, 8, and 9), which was in accord with previous reports (Esposito et al., 2001; Wakao and Benning, 2005). Phylogeny analyses also resulted in the same classification of GmG6PDHs by dividing them into two clades (I and II), with clade I corresponding to P-G6PDHs and clade II corresponding to Cy-G6PDHs (Figure 1A). These results indicated that the divergence of G6PDHs into Cy and P types may be to ensure the plant adaptations to changing conditions.

All the *GmG6PDHs* were found having a close relationship with the corresponding genes in common bean, which is in agreement with their genetic relationships, suggesting possible functional conservation (Figure 1B). Additionally, we found that *GmG6PDHs* had collinear relationships with *S. bicolor*, *O. sativa*, *P. vulgaris* and *B. distachyon G6PDHs*, illustrating that *GmG6PDHs* might have occurred before the divergence of sorghum, rice, common bean and purple false brome grass lineages (Figure 2). The predicted of subcellular localization of plant G6PDHs are basically assigned dependent on the presence of signal peptide (Yu et al., 2004). In the present work, we assessed the subcellular localization of five soybean G6PDHs (GmG6PDH1, 2, 5, 8, and 9) by the transient expression of GFP-tagged recombinant proteins in *Arabidopsis* mesophyll protoplasts (Figure 4). The merged image of chloroplast autofluorescence and GFP indicated that the GmG6PDH1, 5, 8, and 9 fusion proteins were clearly located in the chloroplast, while the GmG6PDH2 protein was specially targeted to cytosol (Figure 4). The results correlated with the *in silico* prediction and phylogenetic clades for GmG6PDHs. The earlier identified AtG6PDH proteins were predicted to be cytosol or plastid-localized isoforms because of the absence or presence of pronounced targeting signal and transmembrane domains (Wakao and Benning, 2005), but a clear experimental evidence was lacking.

Using quantitative real-time qRT-PCR and high-throughput sequencing data analyses, we examined the transcriptional profiling of *GmG6PDHs* in different soybean tissues and at different times during seed development. Our data showed differences in the expression level of *GmG6PDHs* in distinct tissues. To be specific, the plastidic *GmG6PDHs* exhibited a tissue-specific transcript profiling with the high mRNA levels in green tissue, such as leaves and pods (Figure 5A), consistent with the expression profiles of their orthologous genes from *Hevea brasiliensis*, *A. thaliana*, and *Nicotiana tabacum* (Wakao and Benning, 2005; Gong et al., 2012; Long et al., 2016). By contrast, the expression analysis of cytosolic *GmG6PDH* genes revealed relatively high-abundant transcripts in developing seeds, roots and nodules (Figures 5A,B). In *Arabidopsis*, the Cy-G6PDH isoforms are also proved to be crucial in the metabolism of developing seeds (Wakao et al., 2008), suggesting that the cytosolic *G6PDH* genes mainly function in sorts of sink tissues. The tissue-specific expression characteristics indicate that *GmG6PDHs* may play versatile physiological roles in soybean development (Figures 5A–C).

In addition to regulating plant growth, the key functions of plant G6PDHs in stress- response mechanisms have also been widely proven (Esposito, 2016). The promoter regions of the *GmG6PDHs* contained lots of *cis*-acting elements that possibly participated in plant responses to drought and salt stresses, such as MBS, ABRE, ARE, and TCA-elements (Figure 3) (Liu et al., 2017). By qRT-PCR and enzyme activity assays, we further examined the physiological and transcriptional responses of GmG6PDHs to different stress conditions, including salt, alkali and osmotic, and significant induction in *GmG6PDHs* was observed under the above treatments, especially under salt stress (Figures 6, 7). In addition, the different osmotic stresses induced by PEG6000 and mannitol have different effects on the transcription and expression of *GmG6PDHs*, probably due to the inhibitory effects of PEG on root oxygen availability (Guo et al., 2018). It has also been reported that the activities and transcripts of *AtG6PDHs* (Wakao and Benning, 2005), *HbG6PDHs* (Long et al., 2016), *ScG6PDHs* (Yang et al., 2014), and *PsG6PDHs* (Lin et al., 2005) are markedly stimulated by adverse environmental conditions, like oxygen availability, salinity, cold, and dehydration. Furthermore, the subcellular location of *GmG6PDHs* seemed to have certain impacts on their stress responses, as represented by the higher average mRNA expression levels of cytosolic *GmGPDH2*, *4* and *6* in comparison with plastidic *GmG6PDHs* under various stresses. Meanwhile, it is worthwhile to note that a cytosolic isoform (*GmG6PDH2*) respond faster and more vigorously to salinity stress than other genes, reaching its maximum mRNA level (about 100-fold) within 6 h of salinity treatment. The average mRNA abundances of *GmGPDH2* under salt stress was much higher than that of other genes, and its expression patterns correlated well with the activity of G6PDH enzyme (Figure 7), implying a major role in response to salinity. Same results have been available earlier in other plants: a cytosolic G6PDH encoded by *WESR5* are proved to be important for early salt responding in wheat (Nemoto and Sasakuma, 2000, 2002); and a cytosolic *ScG6PDH* also plays a positive role in salt-induced responses in sugarcane, but the corresponding functional verification is lacking (Yang et al., 2014).

In this study, the underlying molecular metabolism of the cytosolic *GmG6PDH2* in mediating salinity adaption was further studied. Enzymatic properties analysis of recombinant GmG6PDH2 proteins expressed in *E. coli* (Figure 8A) showed that the protein encoded by *GmG6PDH2* gene had substrate affinity (*K*_m_^G6P^ of 2.138 mM) (Figure 8C), and a good agreement was achieved compared to the enzymatic characteristics available in previously published G6PDHs (Hauschild and von Schaewen, 2003; Scharte et al., 2009). In addition, overexpression of *GmG6PDH2* dramatically enhanced the salt tolerance in transgenic soybean plants, as reflected by a noticeable increase in root length and root fresh weight (Figures 8E,F). Cytosolic G6PDHs have been previously proved to participate in the modulation of cellular redox states by supply for NADPH (Xu et al., 2003; Valderrama et al., 2006). In the present work, an apparent elevation in the levels of antioxidants (AsA and GSH) as well as the ratios of AsA/DHA and GSH/GSSG were seen in *GmG6PDH2*-OHR plants related to the CHR plants (Figures 9A,B). These data validate that *GmG6PDH2* plays a pivotal role in modulating the changes of metabolite contents and the redox state of AsA and GSH pool under salinity condition, which was consistent with previous results (Krüger et al., 2011; Wang et al., 2016). It is known that the AsA-GSH cycle has a main function of eliminating H_2_O_2_, a potentially harmful ROS (Fotopoulos et al., 2010; Li et al., 2010). In agreement with this, we found that the levels of H_2_O_2_ were obviously lower in *GmG6PDH2* OHRs than in control plants (Figure 9C). In addition, the cellular membrane damage in *GmG6PDH2*-OHR plants was less severe as manifested by the reduction in TBARS content under salt stress (Figure 9D), suggesting that overexpression of *GmG6PDH2* alleviate the salt-induced ROS accumulation and consequently minimize the membrane lipid peroxidation. In summary, the cytosolic *GmG6PDH2* gene has profound effects on salt tolerance by increasing components of the AsA-GSH cycle which subsequently may act to prevent oxidative stress caused by salinity.

## Conclusion

Nine soybean *G6PDH* genes were characterized in the soybean genome. Based on the subcellular localization and phylogenetic analysis, *GmG6PDHs* were divided into plastidic (P) and cytosolic (Cy) isoforms. The *GmG6PDH* genes had distinct expression patterns under various abiotic stresses, reflected the potential functional distinction of each isoform. Of the nine *GmG6PDH* genes, the cytosol-localized GPDH (*GmG6PDH2*) gene had an apparent transcriptional response to salinity, and the expression of *GmG6PDH2* showed a high correlation with the G6PDH activity, suggesting a principal participant in response to salinity. Further study indicated that *GmG6PDH2* gene encode active G6PDH enzyme, which was coordinated with AsA-GSH cycle to maintain the redox state of AsA and GSH and consequently minimize the NaCl-induced oxidative damages.

## Data Availability Statement

All datasets generated for this study are included in the article/Supplementary Material.

## Author Contributions

YZ and YC designed and conceived the experiments. YZ, YC, SH, and JY performed the experiments. XW, DX, XL, YL, and YD analyzed the data and interpreted the results. YZ prepared the manuscript. ZQ and QC conceived the experiments and revised the manuscript. All authors agreed to be accountable for all aspects of the work in ensuring that questions related to the accuracy or integrity of any part of the work are appropriately investigated and resolved and approved the final version to be published.

## Conflict of Interest

The authors declare that the research was conducted in the absence of any commercial or financial relationships that could be construed as a potential conflict of interest.

## Footnotes

1 https://www.soybase.org/sbt

2 http://smart.embl.de/

3 http://pfam.sanger.ac.uk/

4 http://expasy.org/

5 http://www.cbs.dtu.dk/services/TargetP/

6 http://cello.life.nctu.edu.tw/

7 http://gsds.cbi.pku.edu.cn/index.php

8 http://chibba.agtec.uga.edu/duplication

9 https://www.soybase.org/sbt

10 http://bioinformatics.psb.ugent.be/webtools/plantcare/html/

11 https://phytozome.jgi.doe.gov/pz/portal.html

12 https://www.soybase.org/sbt
