# Supplementary figures and images for: Genome-Wide Analysis of the Glucose-6-Phosphate Dehydrogenase Family in Soybean and Functional Identification of GmG6PDH2 Involvement in Salt Stress
Source: Front Plant Sci. 2020 Feb 26;11:214. doi: 10.3389/fpls.2020.00214 (PMC7054389; doi:10.3389/fpls.2020.00214)

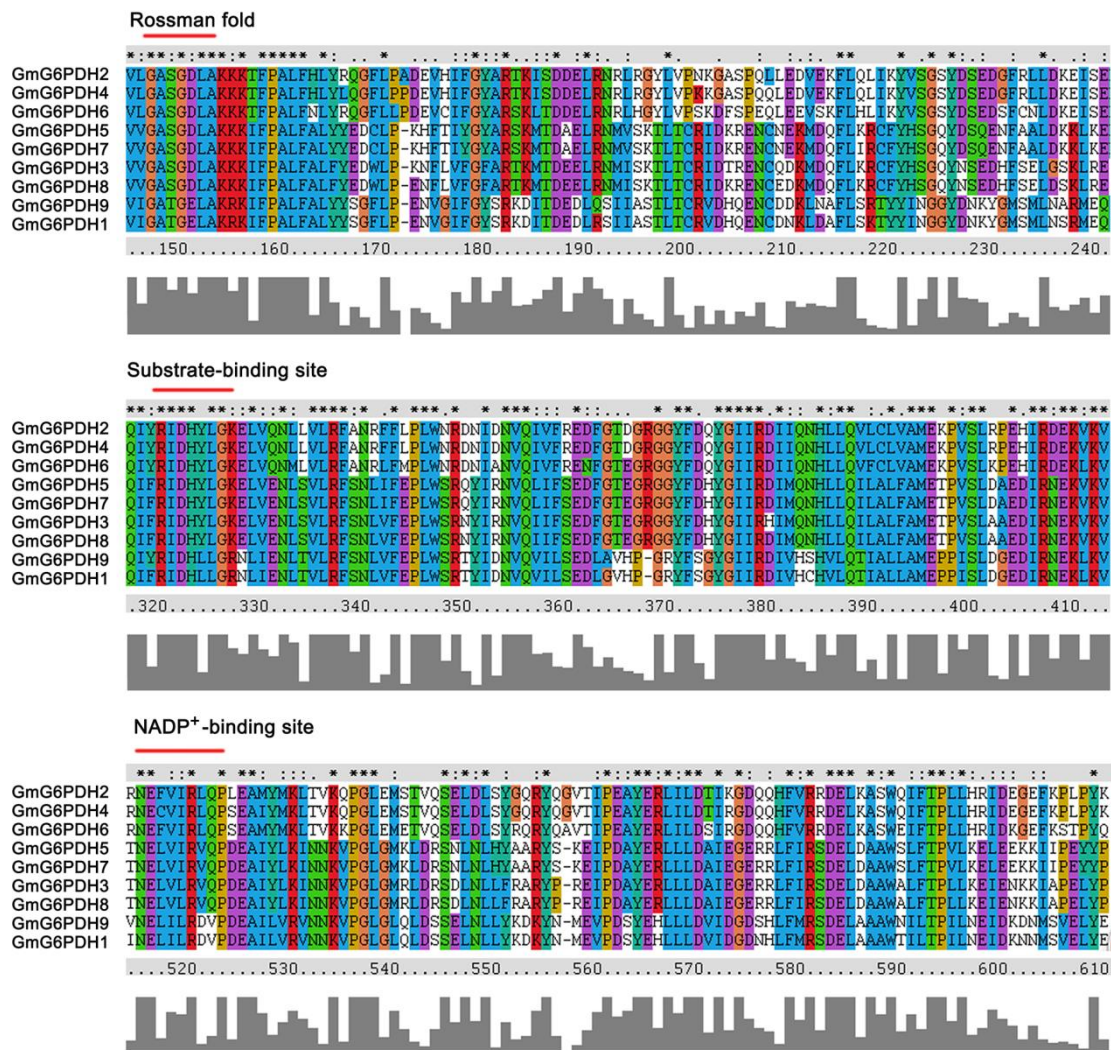

**Figure S1.** Multiple alignment of protein sequences of soybean G6PDHs.

Supplement: Supplementary file 1 [file Image_1.PDF]
