# Supplementary material for: Genome-Wide Analysis of the Glucose-6-Phosphate Dehydrogenase Family in Soybean and Functional Identification of GmG6PDH2 Involvement in Salt Stress
Source: Front Plant Sci. 2020 Feb 26;11:214. doi: 10.3389/fpls.2020.00214 (PMC7054389; doi:10.3389/fpls.2020.00214)
Supplement: Supplementary file 2 [file Image_2.PDF]

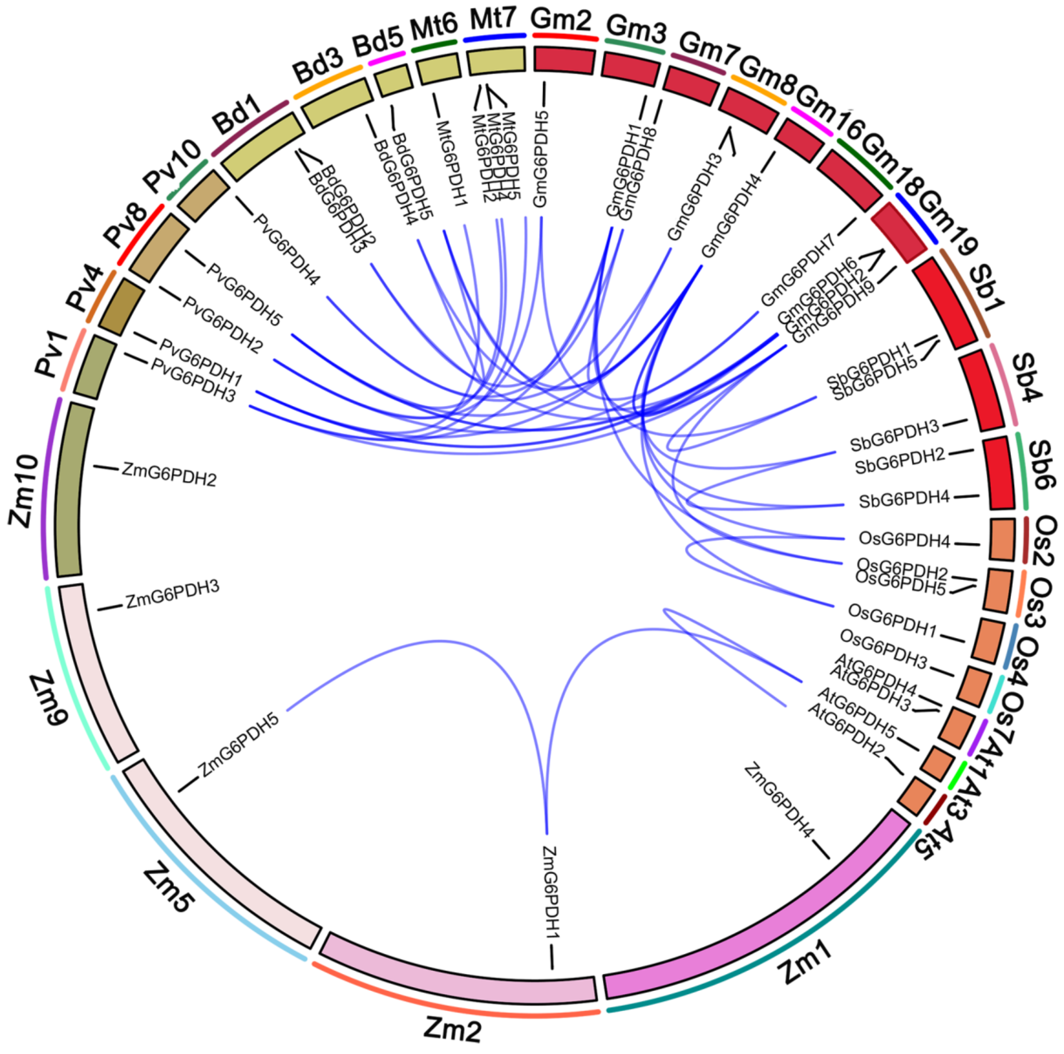

Figure S2. Syntenic analysis of *G6PDH* genes from *G.max*, *Z.mays*, *A.thaliana*, *O.sativa*, *P.vulgaris*, *M.truncatula*, *B.distachyon* and *S.bicolor*.
