# Supplementary material for: Genome-Wide Analysis of the Glucose-6-Phosphate Dehydrogenase Family in Soybean and Functional Identification of GmG6PDH2 Involvement in Salt Stress
Source: Front Plant Sci. 2020 Feb 26;11:214. doi: 10.3389/fpls.2020.00214 (PMC7054389; doi:10.3389/fpls.2020.00214)
Supplement: Supplementary file 3 [file Image_3.PDF]

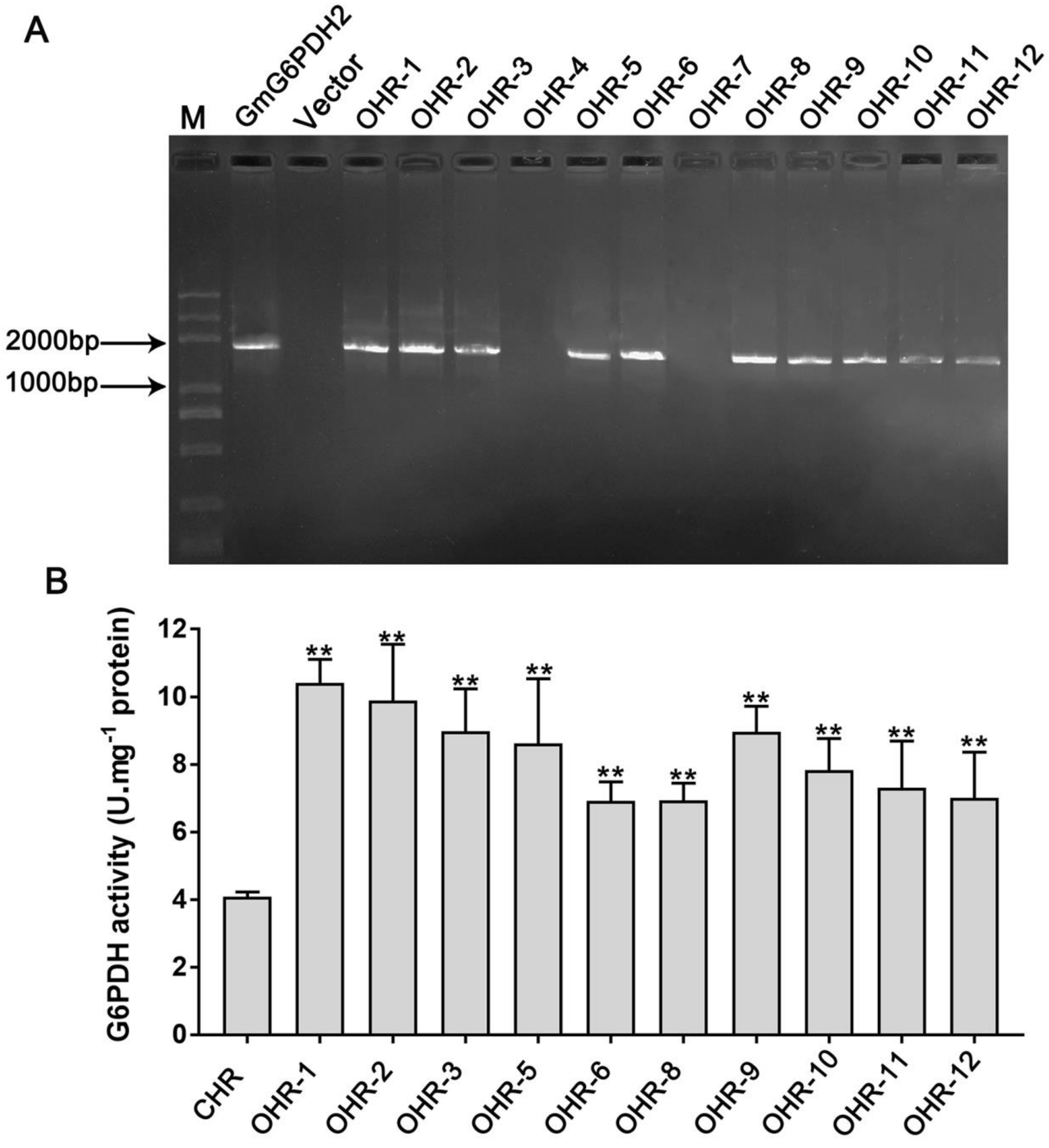

Figure S3. (A) PCR verification of *GmG6PDH2* in the *35s:GmG6PDH2*- overexpressing hairy roots (OHR1-12). M: DNA marker; Vector: the *plasmid* of empty vector (*pBI121-GFP*), as negative control; *GmG6PDH2*: the recombinant expression *plasmid* of *pBI121-GmG6PDH2::GFP*, as positive control. M: DNA Marker DL2000. (B) The G6PDH activities in *GmG6PDH2* transgenic soybean hair root lines (OHR) compared with the CHR. Asterisks indicated significant differences from the CHR, as determined by Student′s t-test (*P<0.05, ** P<0.01).
