# Supplementary material for: Genome-Wide Analysis of the Glucose-6-Phosphate Dehydrogenase Family in Soybean and Functional Identification of GmG6PDH2 Involvement in Salt Stress
Source: Front Plant Sci. 2020 Feb 26;11:214. doi: 10.3389/fpls.2020.00214 (PMC7054389; doi:10.3389/fpls.2020.00214)
Supplement: Supplementary file 4 [file Table_1.DOC]

| **Gene Name** | **Gene ID** | **Gene Location** | | |
| --- | --- | --- | --- | --- |
|  |  | **Chromosome** | **Start** | **End** |
| GmG6PDH1 | Glyma.03G229400.1 | Gm3 | 43144328 | 43150674 |
| GmG6PDH2 | Glyma.19G082300.1 | Gm19 | 29813147 | 29821693 |
| GmG6PDH3 | Glyma.08G199000.1 | Gm8 | 16078525 | 16083556 |
| GmG6PDH4 | Glyma.16G063200.1 | Gm16 | 6210393 | 6217815 |
| GmG6PDH5 | Glyma.02G096800.1 | Gm2 | 8700742 | 8705334 |
| GmG6PDH6 | Glyma.19G077300.1 | Gm19 | 27787739 | 27797138 |
| GmG6PDH7 | Glyma.18G284600.1 | Gm18 | 56525770 | 56534088 |
| GmG6PDH8 | Glyma.07G013800.1 | Gm7 | 1073133 | 1082652 |
| GmG6PDH9 | Glyma.19G226700.1 | Gm19 | 47838450 | 47844119 |
| ZmG6PDH1 | GRMZM2G130230_T01 | Zm2 | 37085168 | 37093582 |
| ZmG6PDH2 | GRMZM2G177077_T01 | Zm10 | 99381158 | 99387758 |
| ZmG6PDH3 | GRMZM2G426964_T01 | Zm9 | 134182737 | 134185295 |
| ZmG6PDH4 | GRMZM2G179521_T01 | Zm1 | 72102454 | 72121802 |
| ZmG6PDH5 | GRMZM2G031107_T02 | Zm5 | 181440754 | 181446379 |
| SbG6PDH1 | Sobic.001G339100.1 | Sb1 | 62704645 | 62707868 |
| SbG6PDH2 | Sobic.006G030800.1 | Sb6 | 6518696 | 6524700 |
| SbG6PDH3 | Sobic.004G204900.1 | Sb4 | 55632561 | 55639949 |
| SbG6PDH4 | Sobic.006G126300.1 | Sb6 | 49163049 | 49169784 |
| SbG6PDH5 | Sobic.001G390900.2 | Sb1 | 67738026 | 67743372 |
| OsG6PDH1 | LOC_Os04g40874.1 | Os4 | 24261369 | 24267801 |
| OsG6PDH2 | LOC_Os03g20300.2 | Os3 | 11468496 | 11475783 |
| OsG6PDH3 | LOC_Os07g22350.1 | Os7 | 12544210 | 12550060 |
| OsG6PDH4 | LOC_Os02g38840.1 | Os2 | 23480215 | 23486812 |
| OsG6PDH5 | LOC_Os03g29950.1 | Os3 | 17067788 | 17072249 |
| MtG6PDH1 | Medtr6g022860.2 | Mt6 | 8007128 | 8015498 |
| MtG6PDH2 | Medtr7g022440.1 | Mt7 | 7240047 | 7247492 |
| MtG6PDH3 | Medtr7g111760.1 | Mt7 | 45900447 | 45906324 |
| MtG6PDH4 | Medtr7g037420.1 | Mt7 | 13788936 | 13791411 |
| MtG6PDH5 | Medtr7g037440.1 | Mt7 | 13798834 | 13806124 |
| PvG6PDH1 | Phvul.004G057000.1 | Pv4 | 7865450 | 7880983 |
| PvG6PDH2 | Phvul.008G016900.1 | Pv8 | 1372056 | 1376747 |
| PvG6PDH3 | Phvul.001G223300.1 | Pv1 | 47839746 | 47845231 |
| PvG6PDH4 | Phvul.010G143200.1 | Pv10 | 42525910 | 42530526 |
| PvG6PDH5 | Phvul.008G148700.1 | Pv8 | 42532949 | 42540782 |
| AtG6PDH1 | AT5G35790.1 | At5 | 13956690 | 13959753 |
| AtG6PDH2 | AT5G13110.1 | At5 | 4158811 | 4161820 |
| AtG6PDH3 | AT1G24280.1 | At1 | 8609445 | 8612580 |
| AtG6PDH4 | AT1G09420.2 | At1 | 3038469 | 3041873 |
| AtG6PDH5 | AT3G27300.1 | At3 | 10083049 | 10086696 |
| AtG6PDH6 | AT5G40760.1 | At5 | 16310748 | 16314774 |
| BdG6PDH1 | Bradi5g13860.1 | Bd5 | 17454410 | 17460517 |
| BdG6PDH2 | Bradi1g64070.1 | Bd1 | 63489784 | 63494745 |
| BdG6PDH3 | Bradi1g60050.1 | Bd1 | 59497774 | 59500954 |
| BdG6PDH4 | Bradi3g47910.1 | Bd3 | 49232505 | 49239269 |
| BdG6PDH5 | Bradi5g04020.1 | Bd5 | 5195919 | 5203683 |
|  |  |  |  |  |

**Table S1.** The gene ID and location of *G6PDH* genes used in this study.
