# Supplementary material for: Genome-Wide Analysis of the Glucose-6-Phosphate Dehydrogenase Family in Soybean and Functional Identification of GmG6PDH2 Involvement in Salt Stress
Source: Front Plant Sci. 2020 Feb 26;11:214. doi: 10.3389/fpls.2020.00214 (PMC7054389; doi:10.3389/fpls.2020.00214)
Supplement: Supplementary file 5 [file Table_2.DOC]

**Table S2.** The primers used in this study.

| **Primer Names** | **Sequence (5'-3')** | **Description** |
| --- | --- | --- |
| GmG6PDH1-F | ATGTCTGTGTCTTTCTCTT | cloning |
| GmG6PDH1-R | TTAATCCTCCACCCAACG | cloning |
| GmG6PDH2-F | ATGGGAACTAGTGAATGG | cloning |
| GmG6PDH2-R | CTACAAGGTAGGAGGGAT | cloning |
| GmG6PDH5-F | ATGGCCACTGCGCTTTAT | cloning |
| GmG6PDH5-R | TTATTGGTCTACGTCTGT | cloning |
| GmG6PDH8-F | ATGGCCAGTATTCTGTGTC | cloning |
| GmG6PDH8-R | TTAGTCTTCGCCAAGATCT | cloning |
| GmG6PDH9-F | ATGTCTGTGTCTTTCTCT | cloning |
| GmG6PDH9-R | CTAATCCTCCACCCATCG | cloning |
| GmG6PDH1-qF | CTTGAGAGACTCAGCTTC | RT-qPCR |
| GmG6PDH1-qR | CCTGTCTGCAGCAACGAT | RT-qPCR |
| GmG6PDH2-qF | ATTGTTGTGCTTGGCGCT | RT-qPCR |
| GmG6PDH2-qR | CTCTACATCCTCCAACAG | RT-qPCR |
| GmG6PDH3-qF | CTCTCATTCACCACTATGG | RT-qPCR |
| GmG6PDH3-qR | CTTAGGTAGCCAATCTTC | RT-qPCR |
| GmG6PDH4-qF | AAGATCTAGCTTCGGCTC | RT-qPCR |
| GmG6PDH4-qR | CTCAATTCATCATCAGAG | RT-qPCR |
| GmG6PDH5-qF | AACGCCTCACCTTCTCAC | RT-qPCR |
| GmG6PDH5-qR | AATACTAACACTGGACTC | RT-qPCR |
| GmG6PDH6-qF | AATGAATCCCCTCTATCG | RT-qPCR |
| GmG6PDH6-qR | ACCTCATCTGGTAGTAGG | RT-qPCR |
| GmG6PDH7-qF | CACCTTCTCACAGCGCTT | RT-qPCR |
| GmG6PDH7-qR | GTCACAGCAGCTGTCACT | RT-qPCR |
| GmG6PDH8-qF | ACCACTATGGCTTTCTTG | RT-qPCR |
| GmG6PDH8-qR | CGGTATATTCTGAATCTG | RT-qPCR |
| GmG6PDH9-qF | TACTTGAGAGACTCAACT | RT-qPCR |
| GmG6PDH9-qR | CCTATGACTGCAATACAA | RT-qPCR |
| GmGAPDH-qF | GACTGGTATGGCATTCCGTGT | RT-qPCR |
| GmGAPDH-qR | GCCCTCTGATTCCTCCTTGA | RT-qPCR |
| GmACTIN4-qF | GTGTCAGCCATACTGTCCCCATTT | RT-qPCR |
| GmACTIN4-qR | GTTTCAAGCTCTTGCTCGTAATCA | RT-qPCR |
| GmG6PDH2-F | CAGTGGTCCCAAAGATGGAC | PCR verification |
| pBI121-GFP-R | CAGATGAACTTCAGGGTCAG | PCR verification |
