# Supplementary material for: Genome-Wide Analysis of the Glucose-6-Phosphate Dehydrogenase Family in Soybean and Functional Identification of GmG6PDH2 Involvement in Salt Stress
Source: Front Plant Sci. 2020 Feb 26;11:214. doi: 10.3389/fpls.2020.00214 (PMC7054389; doi:10.3389/fpls.2020.00214)
Supplement: Supplementary file 6 [file Table_3.DOC]

| **Table S3.** The syntenic relationships among *G.max*, *Z.mays*, *A.thaliana*, *O**.sativa*, *P. vulgaris*, *M. truncatula*, *B. distachyon* and *S.bicolor* *G6PDH* genes. | | | | | | | | | | |
| --- | --- | --- | --- | --- | --- | --- | --- | --- | --- | --- |
| **Block** | ***Arabidopsis thaliana***  **gene location** | | | ***Arabidopsis thaliana* gene ID** | ***Arabidopsis thaliana***  **gene name** | ***Arabidopsis thaliana***  **gene location** | | | ***Arabidopsis thaliana* gene ID** | ***Arabidopsis thaliana* gene name** |
|  | **Chr** | **start** | **end** |  |  | **Chr** | **start** | **end** |  |  |
| 247 | At3 | 10083049 | 10086696 | AT3G27300 | AtG6PDH5 | At5 | 16310748 | 16314774 | AT5G40760 | AtG6PDH6 |
| **Block** | ***Arabidopsis thaliana* gene location** | | | ***Arabidopsis thaliana* gene ID** | ***Arabidopsis thaliana***  **gene name** | ***Zea mays* gene location** | | | ***Zea mays* gene ID** | ***Zea mays* gene name** |
|  | **Chr** | **start** | **end** |  |  | **Chr** | **start** | **end** |  |  |
| 25 | At3 | 10083049 | 10086696 | AT3G27300 | AtG6PDH5 | Zm2 | 37085168 | 37093582 | GRMZM2G130230 | ZmG6PDH1 |
| **Block** | ***Glycine max* gene location** | | | ***Glycine max* gene ID** | ***Glycine max* gene name** | ***Glycine max* gene location** | | | ***Glycine max***  **gene ID** | ***Glycine max***  **gene name** |
|  | **Chr** | **start** | **end** |  |  | **Chr** | **start** | **end** |  |  |
| 1446 | Gm16 | 6210393 | 6217815 | Glyma.16G063200 | GmG6PDH4 | Gm19 | 27787739 | 27797138 | Glyma.19G077300 | GmG6PDH6 |
| 1447 | Gm16 | 6210393 | 6217815 | Glyma.16G063200 | GmG6PDH4 | Gm19 | 29813147 | 29821693 | Glyma.19G082300 | GmG6PDH2 |
| 289 | Gm2 | 8700742 | 8705334 | Glyma.02G096800 | GmG6PDH5 | Gm18 | 56525770 | 56534088 | Glyma.18G284600 | GmG6PDH7 |
| 790 | Gm7 | 1073133 | 1082652 | Glyma.07G013800 | GmG6PDH8 | Gm8 | 16078525 | 16083556 | Glyma.08G199000 | GmG6PDH3 |
| 409 | Gm3 | 43144328 | 43150674 | Glyma.03G229400 | GmG6PDH1 | Gm19 | 47838450 | 47844119 | Glyma.19G226700 | GmG6PDH9 |
| **Block** | ***Oryza sativa* gene location** | | | ***Oryza sativa***  **gene ID** | ***Oryza sativa* gene name** | ***Oryza sativa* gene location** | | | ***Oryza sativa***  **gene ID** | ***Oryza sativa***  **gene name** |
|  | **Chr** | **start** | **end** |  |  | **Chr** | **start** | **end** |  |  |
| 80 | Os2 | 23480215 | 23486812 | LOC_Os02g38840 | OsG6PDH4 | Os4 | 24261369 | 24267801 | LOC_Os04g40874 | OsG6PDH1 |
| **Block** | ***Sorghum bicolor***  **gene location** | | | ***Sorghum bicolor* gene ID** | ***Sorghum bicolor* gene name** | ***Sorghum bicolor***  **gene location** | | | ***Sorghum bicolor***  **gene ID** | ***Sorghum bicolor***  **gene name** |
|  | **Chr** | **start** | **end** |  |  | **Chr** | **start** | **end** |  |  |
| 146 | Sb4 | 55632561 | 55639949 | Sobic.004G204900 | SbG6PDH3 | Sb6 | 49163049 | 49169784 | Sobic.006G126300 | SbG6PDH4 |
| **Block** | ***Zea mays* gene location** | | | ***Zea mays* gene ID** | ***Zea mays* gene name** | ***Zea mays* gene location** | | | ***Zea mays***  **gene ID** | ***Zea mays***  **gene name** |
|  | **Chr** | **start** | **end** |  |  | **Chr** | **start** | **end** |  |  |
| 242 | Zm5 | 181440754 | 181446379 | GRMZM2G031107 | ZmG6PDH5 | Zm2 | 37085168 | 37093582 | GRMZM2G130230 | ZmG6PDH1 |
| **Block** | ***Brachypodium distachyon*  gene location** | | | ***Brachypodium distachyon* gene ID** | ***Brachypodium distachyon*  gene name** | ***Brachypodium distachyon*  gene location** | | | ***Brachypodium distachyon* gene ID** | ***Brachypodium distachyon* gene name** |
|  | **Chr** | **start** | **end** |  |  | **Chr** | **start** | **end** |  |  |
| 184 | Bd3 | 49232505 | 49239269 | Bradi3g47910 | BdG6PDH4 | Bd5 | 17454410 | 17460517 | Bradi5g13860 | BdG6PDH1 |
| **Block** | ***Brachypodium distachyon*  gene location** | | | ***Brachypodium distachyon* gene ID** | ***Brachypodium distachyon*  gene name** | ***Glycine max* gene location** | | | ***Glycine max***  **gene ID** | ***Glycine max***  **gene name** |
|  | **Chr** | **start** | **end** |  |  | **Chr** | **start** | **end** |  |  |
| 801 | Bd5 | 17454410 | 17460517 | Bradi5g13860 | BdG6PDH1 | Gm19 | 29813147 | 29821693 | Glyma.19G082300 | GmG6PDH2 |
| 792 | Bd5 | 17454410 | 17460517 | Bradi5g13860 | BdG6PDH1 | Gm16 | 6210393 | 6217815 | Glyma.16G063200 | GmG6PDH4 |
| 261 | Bd1 | 63489784 | 63494745 | Bradi1g64070 | BdG6PDH2 | Gm19 | 47838450 | 47844119 | Glyma.19G226700 | GmG6PDH9 |
| 32 | Bd1 | 63489784 | 63494745 | Bradi1g64070 | BdG6PDH2 | Gm3 | 43144328 | 43150674 | Glyma.03G229400 | GmG6PDH1 |
| 606 | Bd3 | 49232505 | 49239269 | Bradi3g47910 | BdG6PDH4 | Gm16 | 6210393 | 6217815 | Glyma.16G063200 | GmG6PDH4 |
| **Block** | ***Glycine max* gene location** | | | ***Glycine max***  **gene ID** | ***Glycine max* gene name** | ***Oryza sativa* gene location** | | | ***Oryza sativa***  **gene ID** | ***Oryza sativa***  **gene name** |
|  | **Chr** | **start** | **end** |  |  | **Chr** | **start** | **end** |  |  |
| 737 | Gm16 | 6210393 | 6217815 | Glyma.16G063200 | GmG6PDH4 | Os4 | 24261369 | 24267801 | LOC_Os04g40874 | OsG6PDH1 |
| 728 | Gm16 | 6210393 | 6217815 | Glyma.16G063200 | GmG6PDH4 | Os2 | 23480215 | 23486812 | LOC_Os02g38840 | OsG6PDH4 |
| 884 | Gm19 | 47838450 | 47844119 | Glyma.19G226700 | GmG6PDH9 | Os3 | 11468496 | 11475783 | LOC_Os03g20300 | OsG6PDH2 |
| 124 | Gm3 | 43144328 | 43150674 | Glyma.03G229400 | GmG6PDH1 | Os3 | 11468496 | 11475783 | LOC_Os03g20300 | OsG6PDH2 |
| **Block** | ***Glycine max* gene location** | | | ***Glycine max***  **gene ID** | ***Glycine max* gene name** | ***Phaseolus vulgaris***  **gene location** | | | ***Phaseolus vulgaris* gene ID** | ***Phaseolus vulgaris* gene name** |
|  | **Chr** | **start** | **end** |  |  | **Chr** | **start** | **end** |  |  |
| 606 | Gm8 | 16078525 | 16083556 | Glyma.08G199000 | GmG6PDH3 | Pv10 | 42525910 | 42530526 | Phvul.010G143200 | PvG6PDH4 |
| 1297 | Gm19 | 29813147 | 29821693 | Glyma.19G082300 | GmG6PDH2 | Pv4 | 7865450 | 7880983 | Phvul.004G057000 | PvG6PDH1 |
| 1325 | Gm19 | 29813147 | 29821693 | Glyma.19G082300 | GmG6PDH2 | Pv8 | 42532949 | 42540782 | Phvul.008G148700 | PvG6PDH5 |
| 1115 | Gm16 | 6210393 | 6217815 | Glyma.16G063200 | GmG6PDH4 | Pv4 | 7865450 | 7880983 | Phvul.004G057000 | PvG6PDH1 |
| 1126 | Gm16 | 6210393 | 6217815 | Glyma.16G063200 | GmG6PDH4 | Pv8 | 42532949 | 42540782 | Phvul.008G148700 | PvG6PDH5 |
| 150 | Gm2 | 8700742 | 8705334 | Glyma.02G096800 | GmG6PDH5 | Pv8 | 1372056 | 1376747 | Phvul.008G016900 | PvG6PDH2 |
| 1293 | Gm19 | 27787739 | 27797138 | Glyma.19G077300 | GmG6PDH6 | Pv4 | 7865450 | 7880983 | Phvul.004G057000 | PvG6PDH1 |
| 1331 | Gm19 | 27787739 | 27797138 | Glyma.19G077300 | GmG6PDH6 | Pv8 | 42532949 | 42540782 | Phvul.008G148700 | PvG6PDH5 |
| 1241 | Gm18 | 56525770 | 56534088 | Glyma.18G284600 | GmG6PDH7 | Pv8 | 1372056 | 1376747 | Phvul.008G016900 | PvG6PDH2 |
| 505 | Gm7 | 1073133 | 1082652 | Glyma.07G013800 | GmG6PDH8 | Pv10 | 42525910 | 42530526 | Phvul.010G143200 | PvG6PDH4 |
| 1269 | Gm19 | 47838450 | 47844119 | Glyma.19G226700 | GmG6PDH9 | Pv1 | 47839746 | 47845231 | Phvul.001G223300 | PvG6PDH3 |
| 169 | Gm3 | 43144328 | 43150674 | Glyma.03G229400 | GmG6PDH1 | Pv1 | 47839746 | 47845231 | Phvul.001G223300 | PvG6PDH3 |
| **Block** | ***Glycine max* gene location** | | | ***Glycine max***  **gene ID** | ***Glycine max* gene name** | ***Sorghum bicolor***  **gene location** | | | ***Sorghum bicolor***  **gene ID** | ***Sorghum bicolor***  **gene name** |
|  | **Chr** | **start** | **end** |  |  | **Chr** | **start** | **end** |  |  |
| 588 | Gm16 | 6210393 | 6217815 | Glyma.16G063200 | GmG6PDH4 | Sb6 | 49163049 | 49169784 | Sobic.006G126300 | SbG6PDH4 |
| 584 | Gm16 | 6210393 | 6217815 | Glyma.16G063200 | GmG6PDH4 | Sb4 | 55632561 | 55639949 | Sobic.004G204900 | SbG6PDH3 |
| 703 | Gm19 | 47838450 | 47844119 | Glyma.19G226700 | GmG6PDH9 | Sb1 | 67738026 | 67743372 | Sobic.001G390900 | SbG6PDH5 |
| 74 | Gm3 | 43144328 | 43150674 | Glyma.03G229400 | GmG6PDH1 | Sb1 | 67738026 | 67743372 | Sobic.001G390900 | SbG6PDH5 |
| **Block** | ***Medicago truncatula***  **gene location** | | | ***Medicago truncatula* gene ID** | ***Medicago truncatula***  **gene name** | ***Phaseolus vulgaris***  **gene location** | | | ***Phaseolus vulgaris* gene ID** | ***Phaseolus vulgaris* gene name** |
|  | **Chr** | **start** | **end** |  |  | **Chr** | **start** | **end** |  |  |
| 388 | Mt6 | 8007128 | 8015498 | Medtr6g022860 | MtG6PDH1 | Pv4 | 7865450 | 7880983 | Phvul.004G057000 | PvG6PDH1 |
| 457 | Mt7 | 7240047 | 7247492 | Medtr7g022440 | MtG6PDH2 | Pv8 | 1372056 | 1376747 | Phvul.008G016900 | PvG6PDH2 |
| 408 | Mt7 | 45900447 | 45906324 | Medtr7g111760 | MtG6PDH3 | Pv1 | 47839746 | 47845231 | Phvul.001G223300 | PvG6PDH3 |
| 454 | Mt7 | 13788936 | 13791411 | Medtr7g037420 | MtG6PDH4 | Pv8 | 42532949 | 42540782 | Phvul.008G148700 | PvG6PDH5 |
